# Border Sharpness Index: A Proof-of-Concept Approach for Quantifying Pigmentary Transition Morphology in Vitiligo

**DOI:** 10.7759/cureus.112520

**Published:** 2026-07-12

**Authors:** Jesús Iván Martínez-Ortega, Orson R Juan Hernandez, Sri Naidnur

**Affiliations:** 1 Histology, Autonomous University of Nuevo Leon, Monterrey, MEX; 2 Dermatology, Mexican Institute of Social Security, Piedras Negras, MEX; 3 General Surgery, Mexican Institute of Social Security, Tuxtla Gutiérrez, MEX; 4 Dermatology, Lake Erie College of Osteopathic Medicine, Greensburg, USA; 5 Dermatology, Oasis Dermatology Group, McAllen, USA

**Keywords:** border sharpness index, colorimetry, digital dermatology, digital image analysis, grayscale intensity profiling, lesion border analysis, morphometry, pigmentary transition morphology, vitiligo, wood's lamp

## Abstract

Vitiligo assessment traditionally focuses on lesion extent, depigmentation burden, and disease activity using clinical examination, Wood’s lamp evaluation, and scoring systems, such as the Vitiligo Area Scoring Index (VASI) and Vitiligo Disease Activity (VIDA) score. Although lesion border morphology is routinely recognized during clinical evaluation and may contribute to assessments of disease stability, objective quantitative characterization of pigmentary transition morphology remains largely unexplored. We present an exploratory image-analysis approach based on grayscale intensity profiling of Wood’s lamp images obtained from a 68-year-old woman with vitiligo affecting both hands. Images were converted to 8-bit grayscale using Fiji/ImageJ, and linear intensity profiles were generated across representative lesional borders, healthy skin, and intralesional depigmented areas. To objectively quantify pigmentary transition morphology, we propose an exploratory Border Sharpness Index (BSI) integrating grayscale intensity variation and transition distance. Border-crossing profiles demonstrated reproducible directional intensity transitions that differed from both healthy skin and intralesional control tracings. Healthy skin exhibited minimal physiologic fluctuation, whereas intralesional tracings showed local oscillatory variation without organized directional transition patterns. These findings suggest that grayscale intensity profiling can objectively characterize the spatial architecture of the pigmentary transition zone using standard digital images and freely available image-analysis software. The proposed BSI represents one possible quantitative descriptor of pigmentary transition morphology, integrating morphometric and colorimetric information. While exploratory and nonvalidated, this proof-of-concept methodology introduces a previously underexplored quantitative dimension of vitiligo assessment focused on the spatial architecture of the pigmentary transition zone rather than lesion extent or disease activity alone. Further studies are needed to evaluate reproducibility, clinical correlation, and potential applications in the assessment of lesion stability and therapeutic response.

## Introduction

Vitiligo is a chronic acquired depigmenting disorder characterized by the loss of functional melanocytes and the development of depigmented macules and patches. Beyond its cosmetic manifestations, vitiligo may significantly impact quality of life and often requires long-term monitoring of disease progression, stability, and therapeutic response. Consequently, accurate and reproducible assessment methods are essential in both clinical practice and research settings [1-5].

Several subjective, semi-objective, and objective methods have been developed for vitiligo assessment, including clinical examination, the Vitiligo Area Scoring Index (VASI), the Vitiligo Disease Activity Score (VIDA), the Vitiligo European Task Force assessment (VETFa), digital image analysis, colorimetry, spectrophotometry, and confocal microscopy [1-6]. These approaches primarily focus on lesion extent, depigmentation burden, disease activity, or pigment intensity. Reviews of vitiligo assessment methodologies have emphasized the need for objective, reproducible, non-invasive, and clinically practical tools that integrate both morphometric and colorimetric information [1-4,5,7].

Wood’s lamp examination remains one of the most widely used adjunctive tools in vitiligo evaluation. In addition to facilitating lesion delineation and detection of subclinical depigmentation, Wood’s lamp findings contribute to the assessment of lesion stability and activity. Sharp, well-demarcated borders are generally considered features of stable lesions, whereas ill-defined borders, satellite lesions, and micro-Koebnerization may suggest ongoing disease activity. Nevertheless, interpretation of these morphologic features remains largely qualitative and observer-dependent [6].

Although substantial progress has been made in quantifying lesion extent, pigmentation burden, and disease activity, objective quantitative characterization of pigmentary transition morphology has received remarkably little attention. In particular, spatial transition patterns between lesional and non-lesional skin are routinely recognized by clinicians, yet are rarely evaluated using objective quantitative approaches. The ability to measure such transitions may provide complementary information not directly captured by current scoring systems [1-4,5,7].

In this technical report, we present an exploratory image-analysis approach based on grayscale intensity profiling of Wood’s lamp images using Fiji/ImageJ. We additionally propose a simple Border Sharpness Index (BSI), designed to integrate intensity variation and transition distance as a quantitative descriptor of pigmentary transition morphology. The objective of this work is not to establish a diagnostic or prognostic biomarker, but rather to demonstrate the feasibility of quantitatively characterizing pigmentary transition patterns using readily available digital imaging tools.

## Technical report

Methodology

**Figure 1:**
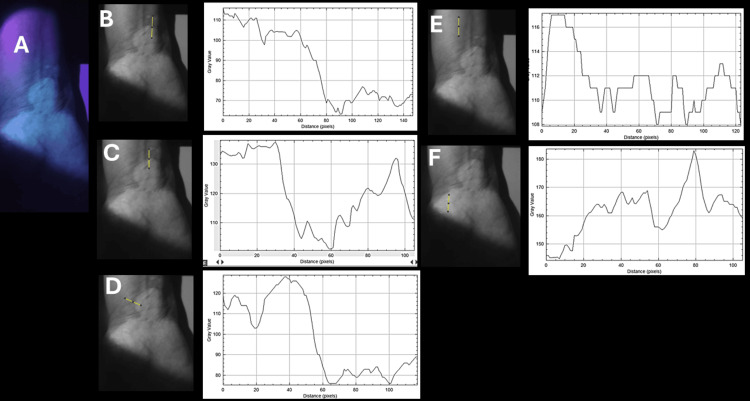
Grayscale intensity profile analysis of vitiligo lesions under Wood’s lamp examination. (A) Representative Wood’s lamp image of vitiligo involving the dorsal hands. (B–D) Three independent linear regions of interest (ROIs) drawn perpendicular to representative lesional borders and their corresponding grayscale intensity profiles, demonstrating sustained directional transitions between lesional and non-lesional skin. (E) Healthy skin control showing minimal physiologic grayscale fluctuation without a structured transition pattern. (F) Intralesional depigmented skin control demonstrating local signal variation without a sustained directional transition. Images were converted to 8-bit grayscale and analyzed using Fiji/ImageJ.

A 68-year-old woman with clinically diagnosed vitiligo involving both hands and an estimated disease duration of approximately three years underwent Wood’s lamp examination as part of routine dermatologic evaluation. Representative lesions were selected for exploratory grayscale intensity profile analysis (Figure 1A).

Wood’s lamp images were analyzed using Fiji/ImageJ software (National Institutes of Health, Bethesda, MD, USA). Images were converted to 8-bit grayscale format before analysis. Linear regions of interest (ROIs) were manually drawn perpendicular to representative lesional borders on grayscale-converted Wood’s lamp images (Figure 1B-D). Additional control tracings were obtained from healthy skin (Figure 1E) and from intralesional depigmented skin without visible border transition (Figure 1F).

For each ROI, grayscale intensity profiles were extracted using the “Plot Profile” function in Fiji/ImageJ, generating sequential intensity values along the traced line. Representative grayscale intensity profiles generated from these tracings are shown in Figure 1B-F. Intensity transitions were qualitatively classified according to the presence or absence of sustained directional signal change.

To explore quantitative characterization of border definition, the grayscale intensity difference was first calculated as

\[ \Delta I = |I_{max}-I_{min}|, \]

where *Imax* and *Imin* correspond to the maximum and minimum grayscale intensities identified along the analyzed intensity profile, respectively.

The transition distance was defined as

\[ d=x_2-x_1, \]

where *x₁ *represents the point at which sustained intensity transition begins and *x₂* represents the point at which the transition stabilizes into a new intensity plateau.

An exploratory parameter termed the BSI was then calculated according to the following formula:

\[ BSI=\frac{\Delta I}{d}, \]

where *ΔI* represents the grayscale intensity difference and *d* represents the transition distance, measured in pixels, between the beginning and end of the major intensity transition zone.

Higher BSI values indicate steeper grayscale transitions occurring over shorter distances and are therefore consistent with sharper pigmentary borders.

Profiles demonstrating abrupt sustained directional intensity changes were interpreted as sharp lesional transitions, whereas profiles showing oscillatory fluctuations without organized directional change were interpreted as intralesional or physiologic variation.

Results

Representative grayscale intensity profiles obtained from Wood’s lamp images demonstrated distinct signal transition patterns between lesional borders, healthy skin, and intralesional depigmented areas (Figure 1, Tables 1-2).

**Table 1:** Qualitative characteristics of grayscale intensity profiles obtained from vitiligo border tracings and control regions. Comparison of signal behavior observed in three independent border-crossing tracings, healthy skin control, and intralesional depigmented skin control. Border-associated profiles demonstrated sustained directional grayscale transitions, whereas control regions lacked organized transition behavior.

Profile	Region analyzed	Dominant pattern	Sustained directional transition	Proposed interpretation
1	Lesional border	Sharp transition	Present	Well-demarcated pigmentary border
2	Lesional border	Sharp transition	Present	Abrupt pigmentary transition
3	Lesional border	Sharp transition	Present	Structured lesional edge transition
4	Healthy skin control	Minimal fluctuation	Absent	Physiologic baseline variation
5	Intralesional control	Oscillatory fluctuation	Absent	Intralesional signal heterogeneity

**Table 2:** Border Sharpness Index (BSI) measurements derived from grayscale intensity profile analysis. Maximum grayscale intensity (Imax), minimum grayscale intensity (Imin), transition distance (d), grayscale intensity difference (ΔI), and calculated Border Sharpness Index (BSI) values obtained from representative vitiligo border tracings and control regions. Higher BSI values indicate steeper grayscale transitions across the analyzed region of interest.

Profile	Δ Intensity	Transition distance (px)	Border Sharpness Index (BSI)
1	42	8	5.25
2	52	10	5.20
3	36	7	5.14
4	9	40	0.22
5	17	35	0.49

Three independent border tracings obtained across representative vitiligo lesion edges demonstrated sustained directional grayscale intensity transitions (Figure 1B-D). These profiles showed abrupt decreases in grayscale intensity followed by stabilization into a new plateau, consistent with sharply demarcated pigmentary borders. Although the exact transition morphology varied between tracings, all border profiles demonstrated organized directional signal change not observed in control regions.

By contrast, the healthy skin control profile (Figure 1E) demonstrated minimal physiologic fluctuation without sustained directional transition, remaining within a relatively narrow grayscale range. Similarly, the intralesional control tracing obtained entirely within depigmented skin (Figure 1F) demonstrated oscillatory local variation without evidence of a structured border-associated transition pattern.

Qualitative pattern classification is summarized in Table 1. Border-associated tracings consistently demonstrated sharp transition behavior with sustained directional intensity change, whereas healthy skin and intralesional controls lacked organized border transition profiles.

Exploratory quantitative analysis further supported these observations (Table 2). Border profiles demonstrated higher BSI values compared with both healthy skin and intralesional controls, reflecting greater grayscale intensity change over shorter transition distances. In contrast, control regions demonstrated low BSI values due to either minimal fluctuation (healthy skin) or broader non-directional oscillatory variability (intralesional tracing).

Collectively, these findings suggest that grayscale intensity profile analysis derived from Wood’s lamp images may provide an objective exploratory approach for characterizing pigmentary border definition in vitiligo.

## Discussion

In this technical report, grayscale intensity profile analysis of Wood’s lamp images demonstrated distinct signal transition patterns among lesional borders, healthy skin, and intralesional depigmented areas. Border-crossing profiles consistently exhibited sustained directional intensity transitions, whereas healthy skin controls showed minimal physiologic fluctuation and intralesional controls demonstrated local oscillatory variation without a structured transition pattern. Collectively, these findings support the feasibility of quantitatively characterizing the morphology of pigmentary transition using simple image-analysis techniques.

Current vitiligo assessment methods primarily focus on lesion extent, disease activity, or overall depigmentation burden. Instruments such as VASI and VETFa provide reproducible measures of affected surface area and pigmentation loss, while VIDA attempts to capture temporal disease activity [1-3,5]. Although these tools have demonstrated reliability and clinical utility, they are not specifically designed to objectively characterize the spatial architecture of the pigmentary transition zone within individual lesions. Consequently, an important morphologic feature routinely recognized by clinicians-the architecture of the pigmentary transition zone-remains largely assessed qualitatively [2-5,7]. To our knowledge, no previously described quantitative parameter has been specifically designed to characterize this feature under Wood's lamp examination objectively.

Wood’s lamp examination occupies a unique position in vitiligo evaluation because it simultaneously facilitates lesion delineation and contributes to clinical assessment of disease stability. Previous studies have reported that sharply demarcated borders are more frequently associated with stable lesions, whereas ill-defined borders, satellite lesions, and micro-Koebnerization may suggest ongoing disease activity. Despite the clinical importance of these observations, border definition is generally described using subjective terminology such as “sharp,” “well-defined,” or “ill-defined,” with limited quantitative characterization [7].

The present exploratory approach attempts to translate this visual perception into a measurable signal-based parameter. Rather than focusing exclusively on lesion area or pigment intensity, grayscale intensity profiling evaluates how pigmentation changes across space. In this context, the proposed BSI incorporates both the magnitude of grayscale intensity variation and the distance over which that variation occurs. Conceptually, this represents a hybrid parameter that integrates colorimetric information (change in signal intensity) and morphometric information (spatial transition distance).

Interestingly, previous reviews of vitiligo assessment methodologies have emphasized the need for objective, reproducible, non-invasive, and clinically practical tools capable of integrating morphometric and colorimetric information. The present method aligns with this objective while remaining technically simple and accessible, relying exclusively on standard digital images and freely available image-analysis software. Although exploratory, this simplicity may facilitate future implementation in larger clinical studies [1-5,7].

Importantly, the purpose of the proposed methodology is not to establish a validated biomarker of disease stability or activity. Rather, it is intended to demonstrate the feasibility of objectively quantifying pigmentary transition patterns that are already recognized during routine clinical assessment. As such, the present work should be viewed as a proof-of-concept methodology introducing a potentially complementary dimension of vitiligo evaluation.

Limitations

Several limitations should be acknowledged. This report represents a proof-of-concept exploratory analysis based on a limited number of manually selected tracings obtained from a single patient. ROI placement and transition point determination were operator-dependent, and no longitudinal clinical correlation, histopathologic validation, or formal reproducibility analysis was performed. In addition, grayscale intensity measurements may be influenced by image acquisition conditions, illumination variability, camera settings, and skin phototype.

The proposed BSI should therefore be interpreted as an exploratory conceptual parameter rather than a validated clinical metric. Further studies involving larger patient cohorts, standardized imaging protocols, and correlation with established measures of disease activity and stability will be necessary to determine its reproducibility, biological significance, and potential clinical utility.

Future perspectives

Future investigations may determine whether quantitative transition parameters correlate with established clinical assessment tools such as VIDA, VASI, VETFa, dermoscopic findings, or Wood’s lamp-based stability criteria. Longitudinal studies could also evaluate whether pigmentary transition profiles change during lesion progression, repigmentation, or therapeutic response. Comparative analyses between clinically stable and unstable lesions may further clarify whether transition morphology contains clinically meaningful information beyond conventional area-based measurements. Future studies may also explore automated extraction of transition profiles from digital images, reducing operator dependence and facilitating integration into automated image-analysis workflows.

More broadly, the concept of quantitative transition analysis introduces a previously underexplored quantitative morphologic dimension that may extend beyond vitiligo to other dermatologic disorders in which border architecture carries diagnostic, prognostic, or therapeutic significance.

The ability to objectively characterize pigmentary transition architecture may therefore represent a valuable addition to the expanding field of quantitative dermatologic image analysis. As quantitative dermatologic image analysis continues to evolve, transition descriptors such as the BSI may serve as complementary morphometric-colorimetric features that can be incorporated into automated image-analysis workflows, machine-learning models, and computer-assisted clinical decision support systems. Integration of transition-based metrics with established clinical scoring systems may further contribute to a more comprehensive and objective evaluation of pigmentary disorders.

## Conclusions

Grayscale intensity profiling of Wood’s lamp images represents a simple and accessible approach for the quantitative evaluation of pigmentary transition morphology in vitiligo. The proposed BSI represents a quantitative descriptor that integrates morphometric and colorimetric information and may provide a complementary dimension of assessment not directly captured by current vitiligo scoring systems. Further studies are required to evaluate reproducibility and clinical relevance in larger patient cohorts.
